# Backcasting the Trust Gap: A Strategic Road Map for Clinician Adoption of AI Diagnostics by 2040

**DOI:** 10.2196/94234

**Published:** 2026-04-30

**Authors:** Yunguo Yu

**Affiliations:** 1 Zyter|TruCare, 2600 Tower Oaks Blvd, Suite 700, Rockville, MD, 20852, United States, 1 6177801330

**Keywords:** medical futures studies, backcasting, trustworthy artificial intelligence, trustworthy AI, small language models, confidence calibration, clinical decision support, futures literacy, health policy, implementation science

## Abstract

The integration of artificial intelligence (AI) into clinical medicine presents a persistent paradox: diagnostic models routinely demonstrate benchmark superiority over human experts, yet bedside adoption remains fragile, and clinician trust is low. Conventional forecasting approaches—projecting model performance along optimistic trend lines—are epistemologically insufficient because they cannot account for the nonlinear sociotechnical transitions that separate technical capability from institutional trust. This Viewpoint applies backcasting, a normative futures methodology with a 4-decade evidence base in energy policy and public governance, to the specific challenge of clinician adoption of AI diagnostics, with the aim of identifying the structural interventions required to achieve durable trust by 2040. Consistent with the tradition of single-expert normative foresight analysis, we applied backcasting as a structured reasoning framework using a STEEP (social, technological, economic, environmental, and political) analysis. Sources from PubMed, IEEE Xplore, Google Scholar, and policy repositories (the US Food and Drug Administration, World Health Organization, Organisation for Economic Co-Operation and Development, and European Commission) published between 2010 and 2025 were reviewed; barriers and enablers were coded across STEEP dimensions to identify pivot points representing convergent, time-bound structural changes. Working backward from a defined 2040 vision state—a health care ecosystem with risk-stratified clinician trust thresholds, semantic transparency of AI outputs, integrated AI governance, and futures literacy in medical education—we identified three temporal pivot points: (1) the 2030 standardization of dual-process AI architectures, in which large language models are verified in real time by locally deployed small language models, producing a calibrated confidence score; (2) the 2035 institutionalization of agentic AI orchestration governed by a formally designated chief AI officer; and (3) the 2040 integration of futures literacy and human-AI teaming competencies into standard medical curricula. The AI trust gap is an institutional design problem, not a technical inevitability. Backcasting reframes the central question from “when will AI be ready for medicine?” to “what must we build to make medicine ready for AI?” The 3 pivot points identified here—verifiable AI by 2030, agentic governance by 2035, and futures literacy by 2040—are structural commitments that clinicians, health system leaders, and policymakers can begin building today.

## Introduction

### Background

The clinical deployment of artificial intelligence (AI)–based diagnostic tools has produced a striking asymmetry: abundant proof-of-concept demonstrations coexist with a persistent deficit of durable bedside adoption. Large language models (LLMs) and deep learning classifiers regularly achieve expert-level performance on curated benchmark datasets, yet surveys of practicing clinicians consistently reveal skepticism, reluctance, and what has been termed “automation resistance” [1]. This is not primarily a technological problem. The bottleneck lies in the sociotechnical substrate: the transparency of AI outputs, the governance structures that certify their safety, the training programs that build clinical fluency, and—above all—the absence of a structured institutional pathway from pilot to practice.

The dominant response to this challenge has been *forecasting*: projecting from current trends in model accuracy, regulatory approval timelines, and computational costs to estimate when “AI will be ready.” This approach is epistemologically inadequate. Forecasting assumes that current trajectories are sufficient conditions for a desired outcome, ignoring the nonlinear policy, cultural, and organizational shifts that are necessary preconditions for deep integration. In complex adaptive systems such as health care, desired futures do not emerge spontaneously from optimistic trend lines; they must be actively constructed through deliberate intervention.

Medical futures studies, a nascent subfield identified and characterized by Meskó et al [2], offers a rigorous alternative. A 2024 scoping review of health care foresight found that only 8 of more than 50 established futures methods are currently applied in health care [2]. This represents a critical underutilization of the foresight evidence base, which has been routinely applied in economics, national defense, and environmental policy for over 4 decades [3].

This Viewpoint applies *backcasting* [4,5] to the specific challenge of clinician AI adoption.

Rather than asking *“*what will happen?*”* backcasting asks “given a desired future, what must happen to get there?” This reframing is essential: the goal is not to predict when AI will be trusted, but to identify the precise structural interventions that will make it trustworthy.

The components of this road map—verification architectures, governance roles, and human-AI teaming (HAT) frameworks—each have precedents in adjacent AI governance and implementation science literature. The contribution of this Viewpoint is not the individual components in isolation, but rather three integrated advances: (1) the use of backcasting as a unifying methodological framework applied specifically to clinician AI adoption, thereby addressing 4 of Dreborg’s [5] canonical conditions for preferring backcasting over forecasting; (2) the identification of 3 pivot points as a dependencies-ordered causal sequence, in which each pivot is a necessary precondition for the next; and (3) the synthesis of dual-process verification, chief AI officer (CAIO) governance, and futures literacy as interlocking necessary conditions rather than parallel independent recommendations.

The aim of this Viewpoint is to apply backcasting as a structured foresight methodology to identify the minimum set of structural interventions required to achieve durable clinician trust in AI diagnostics by 2040.

### Terminology

Throughout this Viewpoint, trust denotes calibrated clinician reliance on AI-generated outputs, as measured by validated automation trust instruments; confidence denotes a model-reported posterior probability estimate; adoption denotes durable clinical use beyond the pilot phase; automation resistance denotes persistent nonuse of AI tools despite demonstrated utility; and semantic transparency denotes the property whereby AI-generated outputs are natively linked to verifiable, computable evidence sources.

## Why Backcasting Is the Appropriate Method

Backcasting was first formalized in energy policy analysis by Robinson [4] as a method for navigating problems where present trends are insufficient, or actively counterproductive, for achieving long-term goals. Dreborg’s [5] canonical definition identifies 4 conditions under which backcasting is the preferred approach over forecasting: the problem is serious, conventional trends are part of the problem, a long time horizon is needed, and dominant interests are implicated. All four conditions apply to the AI trust crisis in medicine:

1. Seriousness—fragmented AI adoption risks entrenching “pilot-phase perpetuity,” a state in which clinical AI tools are perpetually evaluated but never institutionalized, consuming resources without improving outcomes.
2. Current trends as part of the problem—the prevailing trajectory, releasing increasingly powerful LLMs into clinical contexts without any verification infrastructure, increases the rate of hallucination-driven errors, actively eroding the trust that adoption requires [6]. Independent benchmarking of LLMs on clinical tasks has corroborated this concern, documenting systematic calibration failures and overconfident outputs across specialties [7,8].
3. Long time horizon—institutional change in health care (ie, medical education reform, regulatory framework development, and governance role creation) operates on decade-scale timelines, not product release cycles.
4. Dominant interests—technology vendors, health systems, payers, and regulators each hold competing stakes in the current pilot-phase status quo. Only a normative vision of a desired future can align these interests around a common structural agenda.

Backcasting therefore offers a particularly appropriate methodological lens for addressing the AI trust gap in medicine—one aligned with each of the conditions under which Dreborg [5] argues that forecasting alone is insufficient.

## Methodological Execution in This Viewpoint

Consistent with the tradition of single-expert normative foresight analysis, this Viewpoint applies backcasting as a structured reasoning framework rather than as a fully participatory design process. The pivot points were identified through a literature-grounded synthesis executed as follows:

- Sources and timeframe—we searched PubMed, IEEE Xplore, Google Scholar, and policy repositories (the US Food and Drug Administration [FDA], World Health Organization, Organisation for Economic Co-Operation and Development, and European Commission) for publications from 2010 to 2025.
- Search terms—search terms included clinical AI adoption, LLM hallucination health care, confidence calibration clinical decision support, AI governance health systems, CAIO, Futures Literacy medical education, and HAT.
- Social, technological, economic, environmental, and political (STEEP) operationalization—each identified barrier or enabler was coded to ≥1 STEEP dimensions; the 3 pivot points represent the convergence of barriers appearing across multiple dimensions within a shared time horizon.
- Prioritization—no formal Delphi or consensus technique was applied; selection reflects single-expert synthesis and is therefore subject to the perspective constraints acknowledged below.

We acknowledge that formal backcasting, as originally conceived by Robinson [4] and Dreborg [5], typically involves stakeholder cocreation, Delphi consensus, and scenario matrix comparison; those components are outside the scope of this Viewpoint and represent a natural next phase of this research agenda. The vision state, pivot points, and threshold specifications reported here should therefore be interpreted as normative hypotheses requiring multistakeholder validation, not as empirically confirmed targets.

## Defining the 2040 Vision State

The normative foundation of any backcasting exercise is the *vision state*: a precisely specified description of the future toward which the road map is oriented. Vagueness at this stage compromises the entire analysis; the vision must be operationally defined to permit backward inference.

We emphasize at the outset that all threshold values, timeline dates, and structural specifications below are normative design hypotheses requiring multistakeholder validation, not empirically confirmed benchmarks. Their empirical validation is a core task for the formal multistakeholder Delphi process that constitutes the natural next phase of this research agenda.

We define the 2040 vision state as a health care ecosystem characterized by risk-stratified trust thresholds, that is, clinician-reported confidence in *specific AI-assisted diagnostic outputs*—distinct from general willingness to adopt AI—is assessed using validated trust-in-automation instruments adapted for clinical AI contexts [9]. Rather than a single system-wide percentage, the 2040 vision adopts a *risk-stratified trust matrix* in which the required confidence level scales with the clinical task’s autonomy level and potential consequence: for *autonomous task execution* (eg, routine medication reconciliation and simple triage flagging), a trust score of ≥90% is required and for *assistive decision support* (eg, differential diagnosis generation and imaging interpretation), a score of 70% to 85% is acceptable contingent on human-in-the-loop verification. Final threshold values should be determined through specialty-stratified empirical validation and formal stakeholder consensus; if heterogeneous baselines are revealed, the road map may require adaptive pivot timelines.

These ranges are provisional normative anchors, not empirical measurements. Numerical specificity serves a methodological function in backcasting: it constrains the solution space and renders the vision state actionable for governance design. The specific values draw on adjacent automation trust literature [9] and should be replaced by specialty-validated figures as they become available through formal consensus methods. These principles are reflected in the following core dimensions of the proposed vision state:

- Semantic transparency—AI diagnostic outputs are natively linked to verifiable clinical evidence, such that every AI-generated claim carries a computable confidence score grounded in institutional guidelines and peer-reviewed literature.
- Integrated governance—every major health system operates under a formally designated AI governance structure, including a CAIO, with accountability equivalent to the chief medical officer (CMO).
- Futures literacy—medical graduates are trained in HAT and basic foresight competencies as standard components of the clinical curriculum.

This vision state is not utopian. Each element has precedent in an adjacent domain: semantic transparency parallels drug labeling requirements, the CAIO mirrors the chief information security officer trajectory of the 2000s, and futures literacy training already exists in business and public policy education. The question is not whether this state is possible, but what sequence of structural interventions will produce it.

A key concern with backcasting in a rapidly evolving technological domain is whether the vision state end point remains stable across the road map horizon. We address this by defining the vision state at the institutional level—governance structures, curriculum mandates, and trust thresholds—rather than at the technological specification level. Institutional targets are more stable than their underlying technology substrates: they describe what the system must do (verify, govern, and educate), not how it does so. Drug labeling requirements, for example, have survived multiple reformulation cycles without revision to the underlying mandate. We therefore propose an adaptive checkpoint mechanism: at each pivot point, the road map’s technological assumptions should be reviewed against the prevailing state of the art. If those assumptions have materially shifted—for example, if the hallucination problem is addressed by architectural means that make local small language model (SLM) verification unnecessary—the subsequent pivot timelines should be adjusted accordingly, while the vision state’s institutional targets remain fixed.

## Working Backward: 3 Temporal Pivot Points

Backcasting proceeds by identifying *pivot points*—the minimum set of structural changes that, if achieved by a given date, preserve the feasibility of the 2040 vision. We identify 3 such pivot points using a STEEP framework [3] to ensure that each pivot is analyzed across all relevant dimensions.

### The 2030 Pivot: The Verifiable AI Standard

The most proximate—and technically tractable—obstacle to the 2040 vision is the *hallucination* problem: the tendency of LLMs to generate plausible-sounding but factually incorrect clinical claims. Current mitigation strategies (retrieval-augmented generation and post hoc explainability tools) are insufficient because they operate retrospectively and rely on human review as the primary error-catching mechanism [10].

**Figure 1.**
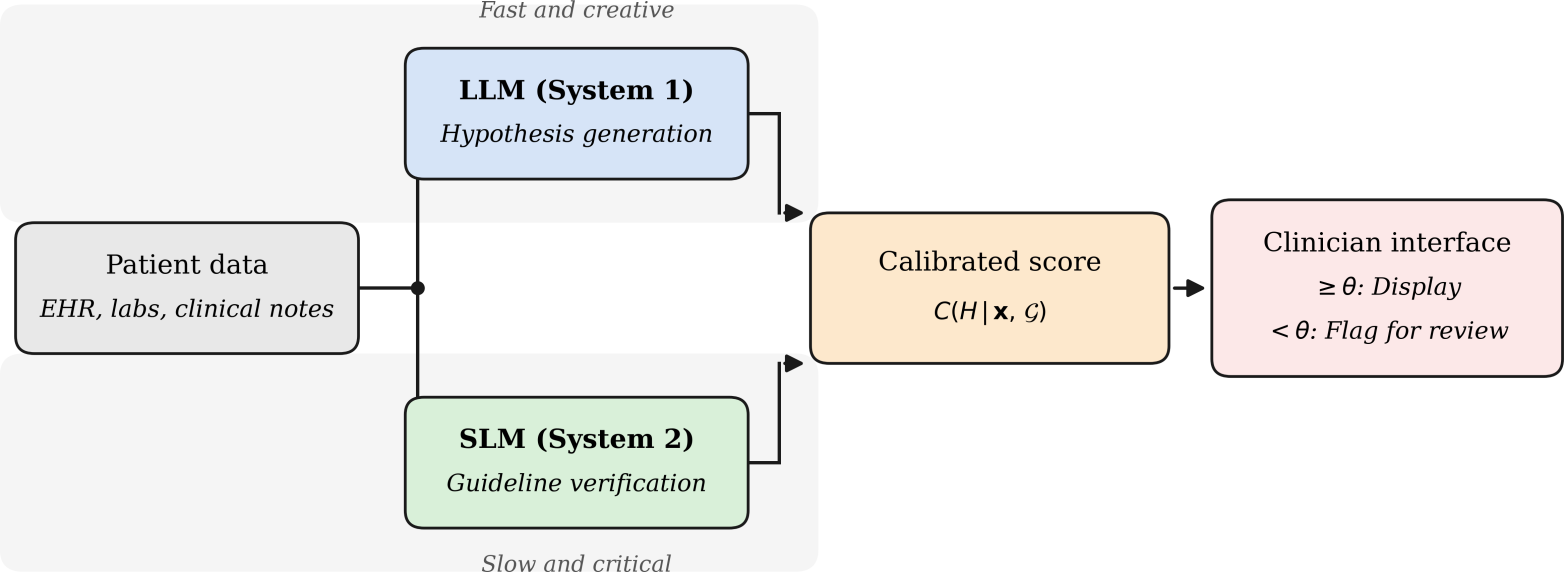
Dual-process artificial intelligence architecture (the “verifiable AI” model)—conceptual framework. Patient data are processed in parallel by a large language model (LLM; generative hypothesis generation) and a locally deployed small language model (SLM; evidence-grounded verification against institutional guidelines *G*). Their outputs are combined into a calibrated confidence score *C(H | x, G)*; claims below verification threshold *θ* are flagged for mandatory clinician review. This figure depicts a proposed conceptual architecture; the confidence equation represents a formal specification requiring prospective validation in operational clinical environments before deployment. Accessibility description: flowchart showing patient data (electronic health record, laboratory results, and clinical notes) entering 2 parallel processing streams. The upper stream feeds an LLM labeled “system 1/fast/creative” for hypothesis generation. The lower stream feeds an on-premise SLM labeled “system 2/slow/critical” for guideline verification. Both streams converge at a calibrated confidence score node; outputs meeting or exceeding threshold *θ* are displayed in the clinician interface, while outputs below *θ* are flagged for mandatory review. EHR: electronic health record.

We propose that the critical technological pivot point for 2030 is the standardization of dual-process AI architectures, conceptually grounded in dual-process theory [11]. This framework includes the following components:

- LLM (system 1)—a general-purpose or fine-tuned LLM generates a rapid, high-breadth diagnostic hypothesis from unstructured clinical data (notes, laboratory results, and imaging reports).
- SLM (system 2)—a domain-embedded SLM, running on local hardware (on premises), cross-references the LLM’s output against a curated institutional guideline corpus *G* and a real-time literature index. It acts as a preceptor, not overriding the LLM but quantifying the evidentiary support for each diagnostic claim. The feasibility of privacy-preserving, redundant local multiagent architectures for clinical tasks has been demonstrated in prototype form [12](Figure 1 shows the 3 components).

The output is a calibrated confidence score, defined formally as follows:


$$C(H|x,G)=P(H|x)\cdot 1\;[S_{SLM}(H,G)\geq \theta ]$$

where *H* is the diagnostic hypothesis; *x* is the clinical input vector; *P(H | x)* is the LLM posterior; *S*_*SLM*_*(H,G)* is the SLM-derived guideline alignment score; *θ* is a system-configured verification threshold; and *𝟙[·]* is the indicator function, equal to 1 when the bracketed condition is satisfied and 0 otherwise. Claims falling below the threshold are flagged for mandatory clinician review rather than being silently passed to the interface. This formulation reflects a gating specification for conceptual clarity; alternative continuous calibration formulations (eg, weighted confidence scaling) may be explored in empirical implementation. Preliminary empirical support for this calibration approach was demonstrated in a validation study of 6689 cardiovascular cases from the Medical Information Mart for Intensive Care-III dataset, in which a dynamic confidence and transparency scoring framework reduced clinician override rates to 33.3%, with high-confidence predictions (90%‐99% confidence) overridden at only 1.7% [13]—representing a 20-fold reduction in override frequency at the highest confidence tier, a finding that suggests clinicians are meaningfully responsive to calibrated confidence signals when they are made explicitly visible. Analogous calibration challenges have been independently documented across diverse clinical AI systems, suggesting that structured verification layers represent a generalizable architectural need rather than a system-specific fix [14,15]. Federated learning frameworks for medical SLMs further demonstrate that locally deployed verification models can be collaboratively trained across heterogeneous institutions—with drift-aware rank scheduling to handle non–independent and identically distributed clinical data distributions—while preserving data locality, providing a practical cross-institutional update pathway for the SLM component of the dual-process architecture [16].

The critical structural condition for this pivot to occur by 2030 is not technological—SLMs capable of clinical guideline alignment already exist in prototype form—but *regulatory*: progressive movement toward formal minimum verification requirements for AI-assisted clinical decision support, analogous to the postmarket surveillance requirements applied to medical devices.

### The 2035 Pivot: The Agentic Orchestration Shift

The 2030 pivot establishes the technical precondition for trust. The 2035 pivot concerns the institutional operationalization of that technical foundation. By 2035, under this road map, AI is envisioned to have evolved from a “consultant” role—passively responding to queries—to an *agentic orchestrator*: autonomously managing longitudinal care tasks such as postdischarge monitoring, medication reconciliation, and chronic disease management workflows [12].

This shift creates governance risks that current health care leadership structures are not designed to manage. Chief information officers (CIOs) lack clinical authority; CMOs lack AI technical literacy. The emerging CAIO role represents the necessary institutional innovation: a formally credentialed position combining clinical expertise, AI technical competency, and governance authority to certify model safety, audit local calibration, and set institutional AI policy.

The CAIO is not intended to replace existing executive roles. Rather, the role bridges the CMO’s clinical accountability and the CIO’s technical infrastructure authority, with specific responsibility for (1) AI model certification and recertification, (2) local calibration auditing and equity reporting, and (3) institutional AI governance policy—functions currently unhoused in either the CMO or CIO role. The CAIO would report jointly to the CMO and the board’s quality and safety committee, with dotted line coordination with the CIO and chief digital officer on technology procurement and data architecture.

The STEEP analysis at this pivot reveals that the primary barrier is not technological but *political*: medical licensing boards, accreditation bodies, and malpractice liability frameworks should be revised to recognize AI-assisted decisions as a category of collaborative clinical output, distinct from both autonomous device operation and unassisted physician judgment [17].

The *economic* and *social* dimensions of this pivot are equally consequential. Economically, the CAIO role and its associated governance infrastructure represent a substantial new cost center for health systems; the business case should be grounded in demonstrable efficiency gains from agentic AI—reduced administrative burden, improved care coordination, and averted adverse events—at a scale sufficient to offset implementation costs. For smaller systems without the resources to sustain a full CAIO function, “CAIO-as-a-service” models offered through regional health authorities or collaborative networks may provide a viable alternative pathway. Socially, the professional identity of the CAIO remains undefined: whether the role is best filled by a clinician with advanced data science training, a computer scientist with a public health qualification, or an entirely new hybrid professional developed through dedicated graduate programs has direct implications for how the 2040 educational pivot should be designed. Accreditation bodies and medical schools should begin addressing this pipeline question within the current planning horizon.

### The 2040 Pivot: Futures Literacy as a Clinical Competency

The final pivot is structural and educational. The 2040 vision state requires clinicians who are not merely passive recipients of AI tools but active participants in shaping the technological environments in which they practice. Integrating futures literacy into medical education requires no new infrastructure; it requires a curriculum decision. Specifically, we propose the incorporation of three elements: (1) scenario analysis into clinical reasoning curricula, replacing some linear differential diagnosis training with probabilistic, multifuture reasoning [18]; (2) HAT modules into clerkship training; and (3) foresight workshops into medical leadership development programs, parallel to the quality improvement and health systems science competencies already mandated by accreditation bodies [19].

## Contingency Logic and Adaptive Pathways

The 3 pivot points above are presented as a dependencies-ordered sequence, but structured backcasting acknowledges that execution may deviate from normative timelines [4,5]. We therefore specify a contingency logic for each pivot. If the 2030 verifiable AI standard is not achieved by the target date—for example, because regulatory standardization is delayed—an interim substitute exists: a voluntary industry consortium standard (analogous to the Health Level 7 Fast Healthcare Interoperability Resources interoperability framework) could provide provisional verification requirements while formal regulation catches up, with the pivot window extended to 2033 and subsequent timelines compressed. If the 2035 agentic governance pivot is delayed, the CAIO-as-a-service model already described provides a distributed interim pathway that does not require mandatory institutional mandates. If the 2040 futures literacy integration has not been achieved, the vision state trust thresholds should be treated as aspirational targets rather than confirmed baselines, and the multistakeholder Delphi process proposed in this Viewpoint becomes the primary recalibration mechanism. At each pivot point, a structured reassessment—rather than abandonment of the vision state—is the appropriate response to schedule deviation.

## The Backcasting Timeline

**Figure 2.**
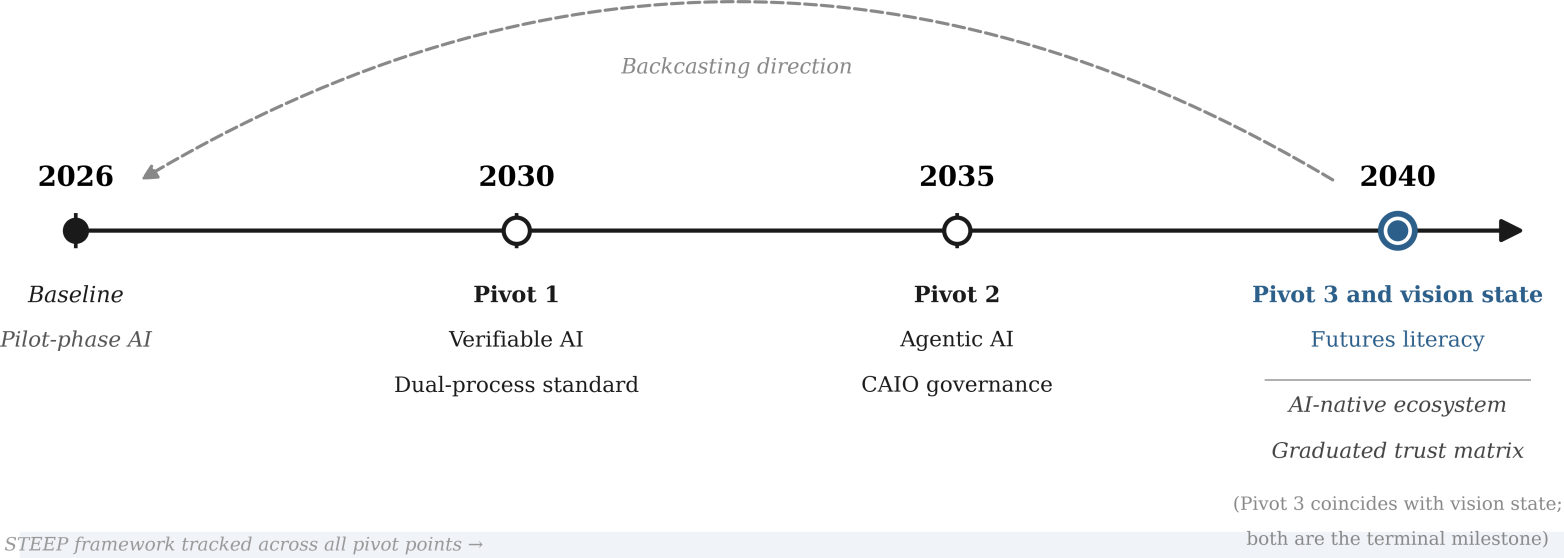
Backcasting timeline: 2026 baseline to 2040 vision state. The dashed arrow represents the normative backcasting direction (vision to present). Open circles mark pivot points where structural interventions should be achieved. The 2040 node carries dual status as pivot 3 (futures literacy) and vision state terminal milestone. AI: artificial intelligence; CAIO: chief artificial intelligence officer; STEEP: social, technological, economic, environmental, and political.

Figure 2 summarizes the 3 pivot points and their relationship to the 2026 baseline and 2040 vision state. Table 1 provides a cross-pivot summary of the required changes across the STEEP framework.

**Table 1.** Temporal milestones across the social, technological, economic, environmental, and political framework.

Dimension	2026 (baseline)	2030 (verifiable)	2035 (agentic)	2040 (vision)
Social	Clinician skepticism and automation fear	SLM^a^ explainability builds baseline trust	AI^b^ fluency enters professional identity	Futures literacy standard
Technological	General LLMs^c^ and no verification layer	Dual-process architecture and on-premises SLM	Agentic orchestrators and ambient monitoring	Seamless XAI^d^ and ambient intelligence
Economic	Pilot budgets and no billing pathways	SLM hardware ROI^e^ established	AI-specific billing codes introduced	System-level AI cost integration
Political	Fragmented and IT-led governance	Regulatory movement toward formal verification requirements	CAIO^f^ mandate and liability frameworks revised	AI-native governance as standard
Environmental	Cloud dependent and high energy cost	On-premise inference reduces footprint	Local edge computing architecture	Distributed, low-latency AI fabric

a SLM: small language model.

b AI: artificial intelligence.

c LLM: large language model.

d XAI: explainable artificial intelligence.

e ROI: return on investment.

f CAIO: chief artificial intelligence officer.

## Discussion

### The Automation Bias Risk

The central finding of this analysis is that the AI trust gap in clinical medicine is not a technical problem awaiting a technical solution, but an institutional design problem amenable to institutional design interventions. Three time-bound structural pivot points—verifiable AI (2030), agentic governance (2035), and futures literacy (2040)—constitute the minimum set of structural changes required to achieve the defined 2040 vision state. The following subsections examine the second-order risks and implementation considerations that the road map itself generates.

Backcasting toward greater AI integration must contend with a well-documented risk: as systems become more reliable, clinicians may exhibit automation bias, accepting AI outputs without appropriate critical evaluation [6]. Systematic reviews of automation bias in clinical decision support have documented its frequency across specialties and identified key mediating factors, including interface design and task complexity [20]. Paradoxically, the more successful the dual-process architecture becomes at reducing errors, the more it may erode the baseline clinical reasoning skills required to function safely during system failures.

The structural response is not to slow AI integration, but to design the verification layer as pedagogically visible. An SLM that surfaces its reasoning—linking each diagnostic flag to a specific guideline citation—functions simultaneously as a safety guardrail and a real-time teaching tool [21]. Critically, this requires a design commitment beyond mere filtering: the SLM should be presented to clinicians as a transparent reasoning partner, not an opaque gatekeeper, so that every interaction reinforces rather than replaces clinical judgment. Longitudinal exposure to SLM-sourced guideline citations can thereby function as embedded continuing medical education, building the domain fluency that the 2040 futures literacy pivot requires. Clinical reasoning and AI collaboration are not zero-sum; they are mutually reinforcing when the verification architecture is made visible.

### Algorithmic Equity and Siloed Bias

The shift from centralized cloud LLMs to locally deployed SLMs introduces a specific equity risk. If an institutional SLM is trained primarily on a single health system’s demographic, it may produce well-calibrated outputs for that population and systematically miscalibrated outputs for underrepresented groups [22]. The CAIO role must therefore explicitly manage the tension between local optimization and national equity benchmarks—a trade-off that will intensify as local models diverge in their calibration profiles across demographically distinct health systems.

We propose 4 concrete structural safeguards. First, an equity audit mandate for the CAIO requires annual recalibration of local SLMs against nationally representative benchmarks, with mandatory public reporting of disaggregated performance metrics stratified by age, race, sex, and socioeconomic status. Enforcement authority would vest in the institutional CAIO, accountable to the same accreditation bodies that currently oversee quality improvement programs. Second, a *cross-institutional calibration exchange*—analogous to federated model benchmarking frameworks piloted in oncology imaging AI [8] and enabled technically by drift-aware federated SLM training [16]—would allow health systems to share calibration data without compromising data sovereignty or patient privacy; participation would require standardized data use agreements modeled on existing federated research network governance (eg, PCORnet). Third, a *public transparency layer* requiring institutions to publish SLM performance statistics across demographic strata would create accountability pressure independent of regulatory enforcement. Fourth, *minimum training data diversity requirements* for locally deployed SLMs—aligned with emerging FDA guidance on AI- or machine learning–based software as a medical device [23]—would establish upstream protections against demographically narrow calibration before deployment. We acknowledge, however, that these safeguards create a structural tension: local optimization for institutional demographics and national equity benchmarks may not be simultaneously achievable, and overly prescriptive diversity mandates risk regulatory fragmentation across health systems. Resolving this tension is a governance research priority for the 2035 pivot.

### Data Sovereignty and Medical Agency

As agentic AI orchestrates longitudinal care decisions autonomously, the current legal framework becomes structurally incoherent: it assigns individual physician liability for all diagnostic and treatment decisions, a structure incompatible with distributed AI-human agency. The road map’s 2035 pivot requires a parallel legal innovation: a framework for *collaborative medical agency* that assigns shared accountability across the human clinician, the institution’s CAIO, and the AI system’s certified governance record.

Aviation’s joint liability structures for automated cockpit systems offer a suggestive—though imperfect—precedent. The analogy has important limitations: aviation operates under a single unified global regulatory body (International Civil Aviation Organization), whereas medicine is governed by fragmented national and regional authorities (FDA, European Medicines Agency, and others) with incompatible approval pathways. Moreover, the aviation cockpit pairs 2 human pilots with a bounded automated system; the clinical “cockpit” may involve 1 clinician simultaneously managing multiple AI agents across care domains.

Despite these differences, several concrete mechanisms from aviation governance translate meaningfully to clinical AI. First, a *decision record log*—analogous to a flight data recorder—could capture the AI system’s inputs, confidence scores, and flagged uncertainties for every consequential clinical recommendation, creating an auditable governance trail for adverse event review. Second, new *institutional malpractice instruments* could name the health system’s CAIO as a coinsured party for AI-assisted decisions, distributing liability in proportion to the documented quality of the institutional governance record rather than assigning it entirely to the attending clinician. Third, a *graduated autonomy protocol*—mirroring aviation’s phased certification of autopilot authority at different flight stages—could specify the conditions under which agentic AI is permitted to act without real-time human confirmation, with those conditions contractually tied to demonstrated calibration performance. Sketching these mechanisms now is speculative; their legal operationalization is, by definition, a task for the 2035 pivot.

### The Parallel Path of Patient Trust

This road map is explicitly clinician centric: its vision state is defined by clinician confidence in AI-assisted outputs, and its pivot points target the technical, institutional, and educational conditions for clinician adoption. However, the ultimate goal of that adoption is improved patient outcomes, and patient acceptance of AI-mediated care is a potentially rate-limiting variable that the road map cannot ignore. A clinician who trusts an AI-generated diagnosis may still encounter a patient who rejects it on the grounds that it originated from a “robot”—a concern with documented precedent in digital health adoption literature [1].

The 3 pivot points each carry patient trust implications. At the 2030 pivot, the semantic transparency requirement—that every AI-generated claim carries a computable confidence score linked to verifiable evidence—could be extended to produce patient-facing explanations: plain-language summaries of why the AI reached a given conclusion, designed to accommodate variability in health literacy. At the 2035 pivot, the CAIO’s governance mandate should include a patient education function: communicating institutional AI policies, audit outcomes, and safeguard mechanisms to patients and communities in accessible formats. By the 2040 vision state, futures literacy in medical education should encompass shared decision-making frameworks for AI-assisted diagnosis, training clinicians to interpret AI outputs collaboratively with patients rather than presenting them as authoritative verdicts. A full account of patient trust is outside the scope of this Viewpoint but represents a necessary parallel research agenda.

### Operational Implementation Barriers

Several operational implementation barriers require explicit acknowledgment beyond the governance and regulatory dimensions already discussed. First, interoperability with legacy electronic health record systems presents a significant technical challenge: locally deployed SLMs must integrate with existing Health Level 7 or Fast Healthcare Interoperability Resources stacks and clinical data repositories through vendor-neutral application programming interfaces, a requirement that presupposes standards that do not yet uniformly exist. Second, workflow burden is nontrivial; a verification layer that introduces perceptible latency at the point of care risks clinician work-arounds that negate its safety function. Third, institutional digital maturity is highly variable: rural and safety-net health systems may lack the compute infrastructure required to run local SLMs, making the CAIO-as-a-service model described above a structural prerequisite rather than an optional alternative for a substantial portion of the health care system. Fourth, medico-legal fragmentation across jurisdictions means that governance frameworks developed for the US regulatory context will require substantial adaptation in the European Union, the United Kingdom, and other settings with different medical device and liability regimes. Fifth, procurement constraints in smaller health systems—where capital budget cycles operate on 5-year timelines—may not align with the decade-scale pivot timelines proposed here; phased procurement schedules and regional consortium purchasing agreements may be necessary to close this gap. Addressing these barriers is a practical prerequisite for the road map’s transition from normative design to operational deployment.

### Limitations

This Viewpoint has 5 principal limitations. First, backcasting is a normative, not predictive, method; the pivot points identified here are necessary conditions for the 2040 vision, not guaranteed outcomes. Second, the 2040 vision state and its risk-stratified trust thresholds (≥90% for autonomous tasks; 70%‐85% for assistive support) reflect expert judgment rather than empirical consensus [13]; future work should establish this vision through a formal multistakeholder Delphi process. Third, this analysis was conducted from a single professional vantage point; patient scholars, frontline nurses, and perspectives from low-resource settings are underrepresented. Fourth, the dual-process architecture requires empirical validation against real clinical datasets to establish whether SLM verification demonstrably improves the calibration of LLM diagnostic outputs. We regard this limitation as a tractable research priority and plan to address it in subsequent work using deidentified clinical data. Fifth, there is a geographic scope limitation: the governance mechanisms, liability frameworks, and accreditation references in this road map are drawn primarily from the US regulatory context (FDA, Accreditation Council for Graduate Medical Education, and state-level malpractice law). Readers in the European Union, the United Kingdom, or other jurisdictions will find the technical and educational pivot points broadly applicable but should adapt the political and legal dimensions to their national regulatory frameworks. A multijurisdictional extension of this road map is a planned next step.

## Conclusions

The trust gap between AI capability and clinical adoption is neither technical nor inevitable. It is an institutional design problem, and it requires institutional design solutions. Backcasting transforms the question from “when will AI be ready for medicine?” to “what must we build to make medicine ready for AI?” The 3 pivot points identified here—verifiable AI by 2030, agentic governance by 2035, and futures literacy by 2040—are not predictions; they are structural commitments that clinicians, health system leaders, and policymakers can begin building today.

Medical futures studies offer the community of practice needed to sustain this work. We invite clinicians, researchers, and AI developers to engage formally with backcasting, scenario planning, and other foresight methods—not as academic exercises, but as essential planning instruments for building institutional capacity to shape health care’s technological future. We additionally propose a multi-institutional consortium to prospectively evaluate the dual-process architecture using deidentified electronic health record data across diverse health systems, with the goal of establishing the empirical pivot evidence base that this normative road map cannot itself provide. The road map offered here is intended as a structured starting point for that institutional work.

## Abbreviations

AI: artificial intelligence
CAIO: chief artificial intelligence officer
CIO: chief information officer
CMO: chief medical officer
FDA: Food and Drug Administration
HAT: human-AI teaming
LLM: large language model
SLM: small language model
STEEP: social, technological, economic, environmental, and political

## References

[1] Topol EJ (2019). High-performance medicine: the convergence of human and artificial intelligence. Nat Med.

[2] Meskó B, Kristóf T, Dhunnoo P, Árvai N, Katonai G (2024). Exploring the need for medical futures studies: insights from a scoping review of health care foresight. J Med Internet Res.

[3] Popper R (2008). How are foresight methods selected?. Foresight.

[4] Robinson JB (1988). Unlearning and backcasting: rethinking some of the questions we ask about the future. Technol Forecast Soc Change.

[5] Dreborg KH (1996). Essence of backcasting. Futures.

[6] Char DS, Shah NH, Magnus D (2018). Implementing machine learning in health care - addressing ethical challenges. N Engl J Med.

[7] Singhal K, Azizi S, Tu T (2023). Large language models encode clinical knowledge. Nature.

[8] Rajpurkar P, Chen E, Banerjee O, Topol EJ (2022). AI in health and medicine. Nat Med.

[9] Jian JY, Bisantz AM, Drury CG (2000). Foundations for an empirically determined scale of trust in automated systems. Int J Cogn Ergon.

[10] Amann J, Blasimme A, Vayena E, Frey D, Madai VI, Precise4Q consortium (2020). Explainability for artificial intelligence in healthcare: a multidisciplinary perspective. BMC Med Inform Decis Mak.

[11] Kahneman D (2011). Thinking, Fast and Slow.

[12] Yu Y (2025). Hybrid-code: a privacy-preserving, redundant multi-agent framework for reliable local clinical coding. arXiv.

[13] Yu Y, Gomez-Cabello CA, Haider SA (2025). Enhancing clinician trust in AI diagnostics: a dynamic framework for confidence calibration and transparency. Diagnostics (Basel).

[14] Guo C, Pleiss G, Sun Y, Weinberger KQ On calibration of modern neural networks. https://proceedings.mlr.press/v70/guo17a/guo17a.pdf.

[15] Moor M, Banerjee O, Abad ZSH (2023). Foundation models for generalist medical artificial intelligence. Nature New Biol.

[16] Yu Y (2026). AdaptiveFedLoRA: drift-aware adaptive LoRA rank scheduling for federated medical small language models. medRxiv.

[17] Price WN, Gerke S, Cohen IG (2019). Potential liability for physicians using artificial intelligence. JAMA.

[18] Wartman SA, Combs CD (2018). Medical education must move from the information age to the age of artificial intelligence. Acad Med.

[19] Miller R (2018). Transforming the Future: Anticipation in the 21st Century.

[20] Goddard K, Roudsari A, Wyatt JC (2012). Automation bias: a systematic review of frequency, effect mediators, and mitigators. J Am Med Inform Assoc.

[21] Miller T (2019). Explanation in artificial intelligence: insights from the social sciences. Artif Intell.

[22] Obermeyer Z, Powers B, Vogeli C, Mullainathan S (2019). Dissecting racial bias in an algorithm used to manage the health of populations. Science.

[23] (2021). Artificial intelligence/machine learning (AI/ML)-based software as a medical device (SaMD) action plan. U.S. Food and Drug Administration.

